# ChIP-Based Nuclear DNA Isolation for Genome Sequencing in *Pyropia* to Remove Cytosol and Bacterial DNA Contamination

**DOI:** 10.3390/plants12091883

**Published:** 2023-05-05

**Authors:** Zehao Zhang, Junhao Wang, Xiaoqian Zhang, Xiaowei Guan, Tian Gao, Yunxiang Mao, Ansgar Poetsch, Dongmei Wang

**Affiliations:** 1 Key Laboratory of Marine Genetics and Breeding (OUC), Ministry of Education, Qingdao 266000, Chinagt_gtian@163.com (T.G.);; 2 College of Marine Life Sciences, Ocean University of China, Qingdao 266000, China; 3 Key Laboratory of Utilization and Conservation for Tropical Marine Bioresources (Hainan Tropical Ocean University), Ministry of Education, Sanya 572000, China; 4 Department of Plant Biochemistry, Ruhr University Bochum, 44787 Bochum, Germany

**Keywords:** bacterial contamination, Chromatin Immunoprecipitation, genome sequencing, histone H3, *Pyropia yezoensis*, plastid DNA contamination

## Abstract

Contamination from cytosolic DNA (plastid and mitochondrion) and epiphytic bacteria is challenging the efficiency and accuracy of genome-wide analysis of nori-producing marine seaweed *Pyropia yezoensis*. Unlike bacteria and organellar DNA, *Pyropia* nuclear DNA is closely associated with histone proteins. In this study, we applied Chromatin Immunoprecipitation (ChIP) of histone H3 to isolate nuclear DNA, followed by high-throughput sequencing. More than 99.41% of ChIP-sequencing data were successfully aligned to the reference nuclear genome; this was remarkably higher than those from direct extraction and direct extraction data, in which 40.96% to 42.95% are from plastids. The proportion of data that were mapped to the bacterial database when using ChIP extraction was very low. Additionally, ChIP data can cover up to 89.00% of the nuclear genome, higher than direct extraction data at equal data size and comparable to the latter at equal sequencing depth. The uncovered regions from the three methods are mostly overlapping, suggesting that incomplete sequencing accounts for the missing data, rather than failed chromatin-antibody binding in the ChIP extraction method. This ChIP extraction method can successfully separate nuclear DNA from cytosolic DNA and bacterial DNA, thus overwhelmingly reducing the sequencing cost in a genome resequencing project and providing strictly purified reference data for genome assembly. The method’s applicability to other macroalgae makes it a valuable contribution to the algal research community.

## 1. Introduction

*Pyropia* is a representative genus in the rhodophyte order Bangiales. It is of fundamental commercial and social importance as the edible seaweed “nori” [1]. *Pyropia* species, particularly *Pyropia yezoensis* (also referred to as *Neopyropia yezoensis*) and *Pyropia haitanensis* (also referred to as *Neoporphyra haitanensis*), are widely cultivated in Asian countries [2,3]. Genetic breeding of new cultivars with improved economic traits, such as higher productivity and better quality, is critical to the commercial development of *Pyropia* spp. as a multibillion dollar, world-wide aquaculture industry [4]. Meanwhile, *Pyropia*, an intertidal red seaweed, has attracted considerable research interest due to its multicellularity, stress adaptation, cellular development, and evolution [5,6]. Genome-associated analysis in *Pyropia* species is required by both genetic dissection of economic traits and basic biological studies. Recently, it was revolutionized by the high throughput and the low cost of next-generation sequencing (NGS) technologies, which have increased the quantity of genome sequencing data [7].

A significant challenge in *Pyropia* genome sequencing is that *Pyropia* species usually have several copies of the chloroplast and mitochondrion genome. When sequencing the data of directly extracted DNA from *P. yezoensis* thalli, chloroplast and mitochondrion genome data can account for up to 70.00% to 80.00% of the total [8,9]. Thus, a vast amount of raw sequencing data is uninformative and more sequencing depth is required for a comprehensive genome analysis. This significantly increases sequencing costs.

In addition to the high abundance of chloroplast and mitochondrial genomes, another issue in *Pyropia* genome sequencing is bacterial contamination. As with other seaweeds, *Pyropia*, are colonized by widely diverse bacteria that interact with them throughout their life cycle [10]. Antibiotic treatments, usually using a mix of several kinds of antibiotics, affect the growth of seaweed and often fail to remove bacteria completely. Researchers used quartz sand polishing to remove bacteria on the algal surface before isolating genomic DNA, but some contamination remained [11]. Once bacterial sequences are included in the data, it is impossible to identify and remove them completely, as the reference genomes for marine bacteria are not sufficient nowadays [10]. Therefore, technology that can avoid bacterial DNA is urgently needed.

Chromatin Immunoprecipitation (ChIP) uses specific antibodies to bind to chromatin and is widely applied to identify protein-associated genomic loci [12]. In eukaryotes, nuclear DNA is tightly bound to a group of basic histone proteins and packaged into a structure called the nucleosome [13,14]. Among the four core histone proteins constituting the nucleosome, histone H3 is highly conserved in eukaryotes [15]. On the contrary, bacterial DNA, as well as the plastid and mitochondrion DNA that originated from ancient bacterial endosymbionts, are naked [16,17]. Nucleoids in bacteria are folded into a range of different conformations by nucleoid-associated proteins (NAPs) that have few similarities in amino acid sequence with histones [18]. In plants, plastid DNA and mitochondrion DNA are anchored with nucleoid-associated proteins (ptNAPs) and high mobility group (HMG) nonhistone proteins, respectively [19,20]. Based on the absence/presence of histone proteins associated with nuclear DNA only, we used the histone H3 antibody in ChIP to specifically isolate nucleosomal DNA from *P. yezoensis* thalli for high-throughput sequencing. We examined the effectiveness of diminishing plastid and bacterial DNA contamination and the genome-wide coverage by comparing the ChIP method to the direct extraction and nuclei extraction methods.

## 2. Results

### 2.1. Histone H3-Based ChIP-Seq Data Collection

We carried out ChIP experiments for *P. yezoensis* thalli and dehydrated thalli using the H3 antibody with two replicated samples. The extracted DNA samples were then used for Illumina sequencing. We also extracted nuclei from each sample using our method to obtain genomic DNA for sequencing. Around 6.5 Gb of Hi-Seq sequencing raw data were obtained for both ChIP-extracted DNA (referred to as ChIP data) and nuclei-extracted DNA (referred to as nuclei data) for each sample (Table 1). After preliminary quality control, about 6.0 Gb of clean data were aligned to the reference nuclear genome of *P. yezoensis*. For ChIP data from both normal and dehydrated thalli, the mapping rates ranged from 99.41% to 99.81%, while for nuclei data, the numbers are lower than 50.00% (47.36–49.91%, *p* < 0.05), but still higher than the one of the direct extraction data (38.76%) (Table 1). To track their possible origin, the unmapped reads were retrieved and aligned to plastid and mitochondrial genomes. More than 81.00% of unmappable reads of nuclei data (corresponding to 40.96% of total clean reads) were successfully mapped onto plastid genomes and 10.00% (corresponding to 5.26% of total clean reads) onto the mitochondrial genome, suggesting that severe contamination with cytosolic DNA still existed after nuclei extraction and that plastid contamination accounted for most of this contamination (Table 1). Among the ChIP data, only 1.00–4.00% of reads unmappable to the nuclear genome were from cytosol DNA (Table 1). The dramatically lower proportion in ChIP data underscores the high efficiency of this method in removing cytosolic DNA contamination in *Pyropia* thalli.

To examine the ChIP method’s capability to remove epiphytic bacterial contamination, we aligned the reads unmappable to nuclear/plastid/mitochondrion to the non-redundant protein sequence database (NR). For nuclei data, 82.00–86.00% of reads (accounting for less than 3.50% of total clean reads) can map to bacterial sequences. The bacterial mapping rate of ChIP data varied from 48.00% to 69.00%, accounting for 0.11–0.40% of total clean reads. For direct extraction data, bacterial contamination increased to 40.68%, accounting for 0.48% of the total clean reads. Although the ratios were not overwhelmingly lower, the practical number of bacterial reads for each ChIP data was only 1/7 or 1/36 (*p* < 0.01) when compared to direct extraction, indicating the effectiveness of the ChIP extraction method in filtering bacterial contamination (Table 1).

### 2.2. Relationship of Transcriptional Activities and Aligned Depth by ChIP Data

**Figure 1:**
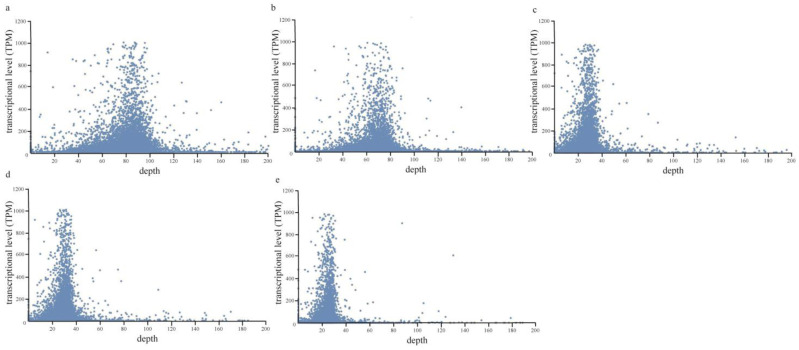
**The scatter diagram shows the relationship between TPM and alignment depth in the different groups.** The groups include ChIP data (**a**), ChIP data (dehydrated) (**b**), direct extraction data (**c**), nuclei data (**d**), and nuclei data (dehydrated) (**e**).

The compactness of DNA and histone proteins affects the accessibility of transcription-related proteins (e.g., transcriptional factors) to DNA and thus determines the transcriptional activity of related genes [21]. Hypothetically, DNA with higher transcriptional activities should have a looser chromatin structure and lower probability of being extracted and sequenced during the ChIP extraction method. To investigate the effect of gene transcriptional activities on its probability of being extracted by the ChIP method, we calculated the aligned depth of each protein-coding gene in the ChIP data of *P. yezoensis* thalli under either normal or dehydrated conditions, and then investigated its correlation to the transcriptional level as detected in the previously published transcriptomic data. Normal distribution patterns were observed in both the normal and dehydrated samples. Genes with low transcriptional levels (TPM < 10) were distributed over a wide range, from zero depth to 1000, while genes with higher transcription had a relatively moderate alignment depth (Figure 1). Similar patterns were also observed in direct extraction data and nuclei data. It appears that there is no direct correlation between the transcriptional activities of genomic loci and their chances to be detected via the ChIP-method; this is inconsistent with our hypothesis.

### 2.3. Genome Coverage of the Three Methods

In addition to the capability of the ChIP extraction method in removing contamination, another concern of this method is whether some genomic regions would be missed due to the incomplete combination of nucleosomes and H3 antibody. To investigate the coverage of ChIP data on the nuclear genome, we investigated the distribution of mappable reads on the reference nuclear genome of *P. yezoensis*, starting with the same amount of clean data produced from the three methods. For ChIP data, 86.00–89.00% of the genome region had at least four reads successfully aligned (aligned depth > 4×). The coverage ratio of corresponding nuclei data was 2.00% to 3.50% lower, and the coverage ratio of direct extraction data was only 79.76%. If we raised the depth threshold to more than 10×, the difference in the coverage ratio between ChIP data and nuclei data was enhanced; this was further enhanced with a threshold of 30×. However, when we randomly selected a portion of clean nuclei data to make the number of mappable reads from ChIP data and nuclei data equal (overall sequencing depth at 21×), the difference in the coverage ratio between the two sets of data declined to less than 1.00%. This suggested that better genome coverage could be achieved by increasing the amount of effective data (mappable data), though it will increase sequencing costs. Therefore, we generated 12.77 Gb of clean ChIP data for one dehydrated thalli sample to enlarge the coverage in the nuclear genome. The genome mapping depth increased to 98× and the coverage ratio increased to 88.12% (1.55% higher than the previous result of 4.0 Gb of data).

**Figure 2:**
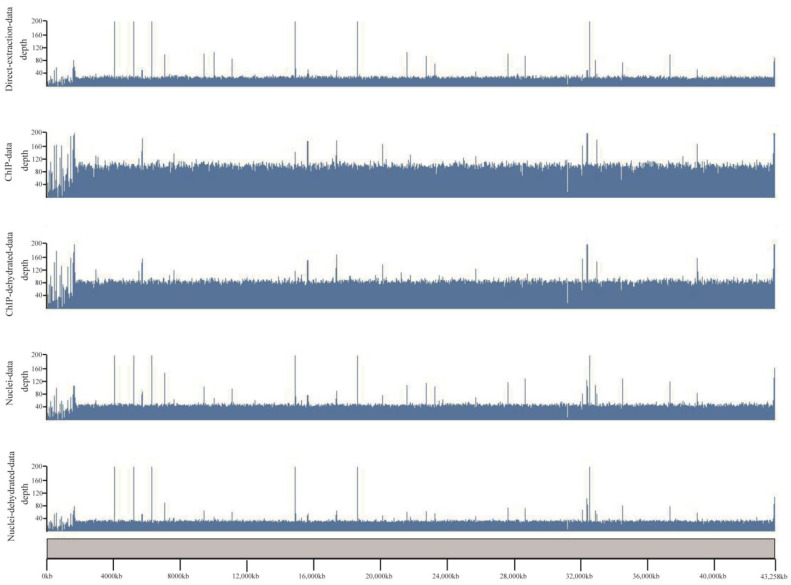
**A visualized distribution of coverage along chromosome 1 for different datasets.** Scale bars of the genome loci were placed at the bottom. The scale on the left side represents the aligned depth of each region.

For a better view of the genomic distribution of covered regions by different datasets, we loaded the alignment results in the karyoplotR program. As shown in Figure 2, except for some ups and downs at the chromosomal end, the covered regions from the three datasets are generally all distributed evenly along chromosome 1, and large missing regions were not observed. We also noticed some peaks in the three datasets. Their patterns in direct extraction data are similar to that in nuclei data, albeit some peaks are apparently missed in the ChIP data.

### 2.4. Features of the Uncovered Region

**Figure 3:**
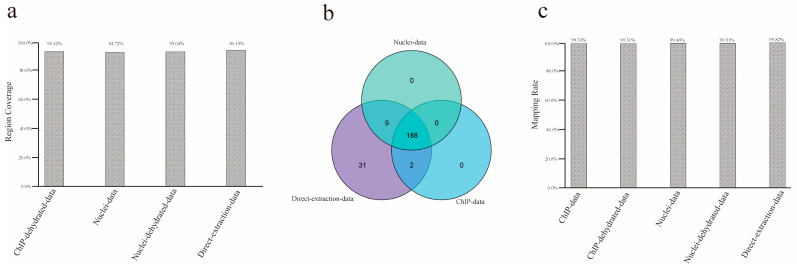
**Features of uncovered regions.** (**a**) Percentages of uncovered regions from ChIP data being successfully aligned by sequences from uncovered regions from nuclei data and direct extraction data, respectively. (**b**) Venn diagram showing the overlapping of uncovered genes. (**c**) Mapping rates of uncovered regions from each method when aligned to PacBio sequencing data.

There are three possible reasons why the above uncovered regions were not detected in sequencing: first, these regions are part of *P. yezoensis* chromosomes and were successfully collected by either of the DNA extraction methods but were too difficult to be sequenced by the Illumina sequencing platform or any platforms due to extreme GC content, high sequence complexity, or other unknown reasons. In this situation, the uncovered regions in each method are supposed to mostly overlap. Second, some regions are difficult to collect using the ChIP extraction method due to the lack of nucleosome structure or low affinity of H3 and its antibody. Therefore, the ChIP method should have generated some specific uncovered regions. Third, some regions in the current reference genome are from bacterial contamination. They were generated in initial genome de novo sequencing and assembly and should also be included in these direct extraction data but less so in direct extraction data and barely in ChIP data. To determine which of the above reason(s) is possible as far as this study is concerned, we retrieved the DNA sequences of the uncovered regions for each method and performed a mutual alignment. About 95.00% of the uncovered regions in ChIP-method overlapped with those in the other two methods (Figure 3a). Then, we collected protein-coding genes linked to these regions; 190, 197, and 230 genes were located within or partially overlapped with the uncovered regions of the combined ChIP data, combined nuclei data, and the 15 Gb of control data, respectively, thus named as uncovered genes of the corresponding method. Interestingly, all the uncovered genes of the combined ChIP data and nuclei data were included in the control group; 188 genes were shared in all three groups, stressing the similarity in uncovered genomic regions in the three DNA extraction methods (Figure 3b). The prominent overlap of the uncovered regions supported the attribution of incomplete sequencing on the Illumina sequencing platform. We observed that about 99.40% of the uncovered regions were successfully aligned by previously reported PacBio sequencing data (Figure 3c).

To further characterize the biological features of the uncovered regions, we first investigated the GC contents. The GC contents of regions not covered by ChIP or direct extraction were about 59.00% and 60.00%, respectively; these were slightly lower than for the nuclear genome (64.63%). N-containing (base unknown) regions accounted for about 3.60–4.60%. About 8000 tandem repeats (TRs, about 0.8 Mb) were identified, and the total length accounted for 5.94–8.34% of uncovered regions; this is comparable to the genome-wide proportion of repeat sequences (Table A1). We then looked at the transcriptional levels and taxonomies of homologs in the NR database of the uncovered genes. Among the 188 shared uncovered genes, 116 (61.70%) exhibited no or scarce transcriptional activity (TPM < 5) in thalli under both normal and dehydrated conditions. In addition to the 21 genes with no blast hits at all, 163 genes showed hits in eukaryotes. Among them, 75 genes had BLAST hits solely in *Porphyra umbilicalis* (another Bangiales genera) and 16 had the best hit in *P. umbilicalis*, followed by other hits in bacteria or eukaryotes, suggesting they were Bangiales-specific genes.

## 3. Discussion

Genome de novo sequencing and re-sequencing of *Pyropia* species suffered significantly from DNA contamination from both epiphytic bacteria and cytosolic organelles, including plastid and mitochondrion. In this study, we developed a ChIP-method to specifically isolate the nuclear DNA that can be subsequently used for the next-generation sequencing.

Compared to the direct extraction and direct extraction methods, this method can overwhelmingly lower the proportion of bacterial, plastid, and mitochondrial data in total sequencing data, without sacrificing genome coverage. As such, this method will make two important contributions: First, it is more economical in terms of sequencing cost and produced data with few extra experimental procedures, as it produces less “garbage” data (bacteria, organelles). Second, it serves as a curation-reference for higher accuracy in de novo genome sequencing and assembly. Due to the high diversity and limited data of marine bacterial sequences, even a small amount of nuclei data during the assembly will bring about severe contamination; this is because it is currently impossible to distinguish between and remove them afterwards. This ChIP method can significantly reduce the content of bacterial DNA and generate “clean” DNA for both the Illumina short-reads sequencing platform and long-reads platforms such as PacBio. For genomic regions and genes in a finished genome assembly, being successfully aligned by ChIP-extracted “clean” data will provide strong evidence that the genes are free of contaminants. Moreover, the ChIP extraction method can also be applied to other organisms that are suffering from bacterial or plastid DNA contamination, especially marine seaweeds such as *Gracilaria*, *Bangia*, and others.

We failed to obtain sequencing data for more than 10.00% (17.66 Mb) of regions in the *P. yezoensis* genome using the ChIP method. Most of these data were also not detected by either the direct extraction method or the direct extraction method, suggesting that incomplete sequencing accounts for the missing data. This hypothesis is supported by a higher coverage ratio when increasing the sequencing depth. For further improvement, combined antibodies of histones H3 and H4 to increase affinity to the nucleosome, mixed biological samples from different developmental stages or stress treatments to exclude the effect of transcriptional activities on chromatin structure, and improved experimental procedures of the ChIP protocol, such as CUT & Tag [22], should be helpful for achieving higher genome coverage.

Previous ChIP-Seq analysis for a transcription factor or the histone modification mark in animals and plants revealed false positive genomic regions and enrichment of high-affinity binding sites [23,24]. Sequencing biases, such as GC-rich regions, also exist in ChIP-Seq [25]. In this study, although the overall genomic distribution of the covered region for each DNA extraction method was similar, we noticed varied sequencing depths in certain genomic regions. Since sequencing data were generated by the same platform, we believe the main cause of this variation lies in the different accessibility and affinity of antibodies to histone H3, which is dictated by varying chromatin structure.

Additionally, *P. yezoensis* harbors epiphytic fungi, such as ascomycetes and oomycetes, on marine seaweeds [26,27]. Histone proteins are the key players in separating nuclear DNA from cytosolic and bacterial DNA in the ChIP method. Since histones H3 and H4 also exist in fungi with well-conserved protein sequences, it is possible that our ChIP method cannot distinguish this fungal DNA.

## 4. Materials and Methods

### 4.1. Algal Culture and Sample Collection

The thalli of *Pyropia yezoensis* (accession GCA_009829735.1) were used in this study. The thalli were cultivated in boiled natural seawater with Provasoli’s enrichment solution medium (PES) at 10 °C in an incubator (GXZ-280B, Jiangnan Instrument Factory, Ningbo) with a light concentration of 20 μmol photons·m^−2^·s^−1^ and a 12/12-h light/dark cycle [28]. The medium was refreshed every three days. Thalli at the age of 45 days were used for genomic DNA extraction. Fresh thalli were collected directly from the culture medium. Surface water was removed using autoclaved gauze, and the samples were immediately frozen in liquid nitrogen. They were then immediately frozen in liquid nitrogen for direct extraction or direct extraction of genomic DNA or put into 1.00% formaldehyde solution for ChIP extraction (detailed procedures are described below). For dehydration treatment, wiped thalli were placed in a glass dish and exposed to air in an incubator. They were collected when their absolute water content (AWCs) reached 50.00%, as described in Mao et al. [29]. Two biological replicates were collected for each genomic DNA extraction method.

### 4.2. Chromatin Immuno-Precipitation (ChIP) and Hi-Seq Sequencing

ChIP experiments were performed following the procedures described by Wei et al. in 2018 [30]; the detailed protocol is shown in the supplementary material (File S1). Briefly, at 45 days of age, the thalli were collected and treated with 1.00% formaldehyde for DNA/histone cross-linking for 30 min. After quenching the cross-linking reaction with 2M glycine, we froze the cross-linked thalli samples with liquid nitrogen and ground them. Chromatin was isolated by sucrose density gradient centrifugation. Histone H3 antibody (ab176842, Abcam, Cambridge, UK) and Magna ChIP™ Protein A+G Magnetic Beads (16–663, Sigma-Aldrich, St. Louis, MO, USA) were used for immunoprecipitation in this study. ChIP DNA was purified using the Universal DNA Purification Kit (DP214-02, Tiangen). The purity and integrity of ChIP-extracted DNA were analyzed using agarose gel electrophoresis. The concentrations of ChIP DNA samples were quantified by Qubit. Paired-end sequencing libraries were prepared, as instructed by the Illumina standard protocol, and then loaded on the Illumina HiSeq 2000 platform for sequencing. For one of the dehydrated samples, we generated approximately double the amount of ChIP data (about 13.7 Gb) in order to investigate the effect of data size on genome coverage.

### 4.3. ChIP-Seq Data Analysis

Raw data were trimmed by Fastp to obtain the clean data [31]. Quality control statistics were generated using FastQc [32], and clean data were mapped to the *Pyropia yezoensis* genome (GCA_009829735.1) and gene database using BWA [33]. Sorting alignment by the 5′ position on the chromosome and filtering out alignments with low mapping quality were performed using samtools [34]. Depth and coverage were counted by bamdst (https://github.com/shiquan/bamdst, accessed on 19 February 2023). Covered regions were plotted by karyoplotR [35]. VEN-plots were performed on TBtools [36]. Tandem repeats were identified using by the Tandem Repeats Finder (https://tandem.bu.edu/trf/trf.html, accessed on 20 October 2022). Randomly picking reads from a dataset was performed by seqtk (https://github.com/lh3/seqtk, accessed on 17 February 2023).

Transcriptomic data of dehydration samples were downloaded from a previously published work [37]. The gene expression level was measured by TPM (Transcripts per kilobase of exon model per million mapped reads) and calculated using stringtie [38,39].

### 4.4. Genomic DNA Preparation by Direct Extraction or Nuclei Extraction

*Pyropia yezoensis* thalli were frozen in liquid nitrogen and ground into a powder. Then, DNA was isolated following the instruction of the one-step-lysis plant DNA kit (Nobelab, Beijing, China). This approach is referred to as direct extraction. For nuclei extraction, thalli were frozen in liquid nitrogen and ground. Nuclei were then isolated by sucrose density gradient centrifugation, as described by Zhang et al. [40], followed by DNA purification using the Universal DNA Purification Kit (DP214-02, Tiangen, Beijing, China).

### 4.5. Statistical Analysis

The data were analyzed using the statistical software SPSS Statistics 25.0 for Windows. Independent-samples *t*-tests were applied to examine the significance.

## Figures and Tables

**Table 1:** Statistics of sequencing data and mapping rates.

	Raw Data (Gb)	Clean Data (Gb)	Nuclear Mapping Rate	Plastid Mapping Rate	Mitochondrion Mapping Rate	Bacteria Mapping Rate
ChIP data1 *	7.95	7.32	99.81%	0.0055%	0.0014%	0.11%
ChIP data2	8.12	7.54	99.41%	0.0087%	0.0016%	0.40%
ChIP-dehydrated data1	13.71	12.77	99.76%	0.0049%	0.0010%	0.14%
ChIP-dehydrated data2	7.32	6.80	99.62%	0.012%	0.0019%	0.18%
Nuclei data1	8.10	7.83	49.91%	40.96%	5.26%	3.27%
Nuclei data2	6.14	6.20	47.36%	42.95%	5.42%	3.52%
Nuclei dehydrated data1	11.10	10.72	48.63%	42.00%	5.37%	3.42%
Nuclei dehydrated data2	6.39	6.11	49.21%	41.18%	5.42%	3.51%
direct extraction data	11.20	10.70	38.76%	53.41%	6.64%	0.48%

* data1 and data2 of each method were from the two biological replicates.

## Data Availability

Data in this work it is available on reasonable request, please contact Z.Z. at skyzhangzehao@163.com or D.W. at wangdm@ouc.edu.cn.

## Supplementary Materials

The following supporting information can be downloaded at: https://www.mdpi.com/article/10.3390/plants12091883/s1, File S1: Detailed protocol of ChIP-based DNA isolation in *Pyropia*.

## Appendix A

**Table A1:** GC contents, N contents and tandem repeat contents of uncovered regions in the three methods.

	Full Length	GC	N	TR Number	TR Length	TR Length Rate
ChIP data	10,901,248	59.05%	4.60%	6162	684,452	6.28%
ChIP dehydrated data	10,777,313	59.03%	4.65%	6203	677,089	6.28%
nuclei data	12,013,931	60.37%	4.17%	9668	950,651	7.90%
nuclei dehydrated data	11,968,968	59.92%	4.19%	8643	901,127	7.52%
direct extraction data	13,901,143	61.67%	3.60%	14,039	1,255,462	9.03%
