# Supplementary material for: ChIP-Based Nuclear DNA Isolation for Genome Sequencing in Pyropia to Remove Cytosol and Bacterial DNA Contamination
Source: Plants (Basel). 2023 May 5;12(9):1883. doi: 10.3390/plants12091883 (PMC10181236; doi:10.3390/plants12091883)
Supplement: Supplementary file 1 [file plants-12-01883-s001.zip › Supplementary File-ChIP- based nuclear DNA isolation for genome sequencing in Pyropia to remove bacterial and cytosol DNA contamination-protocal.pdf]

Detailed protocol of ChIP-based DNA isolation in *Pyropia*.

1. Materials

The thallus of *pyropia yezoensis* (accession GCA_009829735.1) was used in this study. Thallus were grown in boiled natural seawater with Provasoli's enrichment solution medium (PES) at 10 °C in incubator, with a light con-centration of 20μmol photons · m^{-2} · s^{-1} and a 12/12-h light/dark cycle. The medium was refreshed every three days.

2. Reagents

2.1 Antibodies

Anti-Histone H3 antibody (ab176842; Abcam), which is a rabbit monoclonal antibody for purification of DNA binding with histone H3.

2.2 Chemicals

Formaldehyde solution; Glycine; Magnesium chloride hexahydrate (MgCl2•6H2O); Sucrose; sodium hydroxide (NaOH); EDTA-2Na; Triton X-100 (T8200, solarbio); 2-Hydroxy-1-ethanethiol (β-ME); Tris-HCl (PH=8.0) (T1150, solarbio); 10%SDS (S1010, solarbio); sodium chloride (NaCl); cOmplete, EDTA-free, EASYpack (4693132001, ROCHE); Protease K (P1120, solarbio); RNase A Solution (10mg/ml) (R1030, solarbio);

2.3 Kit

The Universal DNA Purification Kit (DP214-02, Tiangen).

2.4 Stock solutions

- 1M magnesium chloride ($MgCl_2$)
- 2M sucrose
- 2M glycine
- 0.5M EDTA-2Na (PH=8.0)
  Note: 0.5M NaOH is used to dissolution EDTA-2Na.
- 1M sodium chloride (NaCl)
- 5M sodium chloride (NaCl)
- 10% Triton X-100
- 2.5X protease inhibitor cocktail
  Note: a slice of cOmplete, EDTA-free, EASYpack is dissolved in 2ml double distilled water.

2.5 Working solutions

- Crosslinking buffer (1% Formaldehyde solution)
- Extraction buffer 1 (10mM $MgCl_2$, 0.4M Sucrose, 10mM Tris-HCl (PH 8.0), 5mM β-ME)

- Extraction buffer 2 (10mM MgCl2, 0.25M Sucrose, 10mM Tris-HCl (PH 8.0), 5mM β-ME, 1% Triton X-100, 1X protease inhibitor cocktail)
- Extraction buffer 3 (2mM MgCl2, 1.7M Sucrose, 10mM Tris-HCl (PH 8.0), 5mM β-ME, 0.15% Triton X-100, 1X protease inhibitor cocktail)
- Lysis Buffer (50mM Tris-HCl (PH 8.0), 10mM EDTA-2Na (PH=8.0), 1% SDS, 1X protease inhibitor cocktail)
- ChIP dilution buffer (1% Triton X-100, 1.2mM EDTA-2Na (PH=8.0), 1X protease inhibitor cocktail, 16.7mM Tris-HCl (PH 8.0), 167mM NaCl)
- Low-salt washing buffer (20mM Tris-HCl (PH 8.0), 150mM sodium chloride (NaCl), 0.1%SDS, 1% Triton X-100, 2mM EDTA-2Na (PH=8.0))
- High-salt washing buffer (20mM Tris-HCl (PH 8.0), 500mM sodium chloride (NaCl), 0.1%SDS, 1% Triton X-100, 2mM EDTA-2Na (PH=8.0))

2.6 Equipment

Eppendorf microcentrifuge tubes; Hitachi CR21N High-Speed Refrigerated Centrifuge; Covaris S220 Focused Ultrasonicator, AFA, Covaris S2, M220, DNA Shearing; microtube AFA Fiber Pre-Slit Snap-Gap (6x16mm, 520045, Covaris); Magna ChIP™ Protein A+G Magnetic Beads (16-663, Sigma-Aldrich); Qubit 2.0 Fluorometer (Invitrogen).

3. Experimental Protocol

- **Cross-linking**

a) Take 0.2g of material, add it into 50ml centrifuge tube containing 37ml crosslinking buffer, and rotating at room temperature for 30min.
b) Add 2.5ml glycine (2mol/L) and quenching crosslink at room temperature for 10min.
c) Rinse with clean seawater for 3 times, wipe dry with paper towel and freeze with liquid nitrogen. If samples need to be stored, they should be stored at - 80 °C (2 months at most).

- **Chromatin extraction (unless otherwise specified, all samples shall be placed on ice for operation)**

a) Grind the cross-linked cells to fine powder in liquid nitrogen for 5 min using a mortar and pestle.
b) After grinding the sample with liquid nitrogen, transfer it to a centrifugal tube containing 25ml buffer 1, place it on ice and turn it upside down for 30min.
c) Filter into a new centrifuge tube with a 100um filter.
d) Centrifuge 1500g at 4 °C for 20min.
e) Discard the supernatant as much as possible, add 1ml buffer 2 to resuspend the pellet, blow and mix with a pipette, and then transfer it to a 1.5ml EP tube.
f) Centrifuge 16000g at 4 °C for 10min.
g) Discard the supernatant as much as possible, prepare a clean Eppendorf tube with 500µL of Extraction Buffer 3. Take the 450µL solution (resus-pended pellet) from last step and carefully layer it on top of the clean tube. Centrifuge the sample for

1h at 16,000g at 4°C.

- **Sonication**

a) Discard the supernatant, resuspend the pellet again in 1ml lysis buffer, blow it up and down. Another 25μL is stored at - 20 ℃ as a control for identification of sonication.
b) S220 is used for ultrasonic interruption, and the setting conditions are: 105 peak power; 2 duty factor; 200 cycles per burst; 18min treatment time; 3-9℃ temperature. Collect the broken chromosome into a new EP tube.
c) Centrifuge 1500g at 4 ℃ for 5min.
d) Take the supernatant into a new EP tube. Take 25μL for identification of sonication. Take 10-50μL as nuclei. Keep the rest at - 80 ℃.

- **Assay of sonication**

a) Prepare 25μL no-sonicated samples (step sonication-a) and 25μL sonicated samples (step sonication-d).
b) Add 2μL NaCl(5M), 1μL Proteinase K and 22μL lysis buffer to the two samples to a total volume of 50ul. Incubate overnight at 65 ℃.
c) DNA Purification Kit is used to recover DNA.
d) using agarose (1%-2%) gel electrophoresis to carry out identification of sonication. The sonicated DNA fragment size should be 250-750bp.

- **Antibody incubation and immunoprecipitation**

a) Take 100μL of recovered chromatin and dilute it to 10 times of the original with chip dilution buffer.
b) 5μL Histone H3 antibody was added to the diluted sample. Incubate at 4 ℃ for overnight.
c) Gently upside down and resuspend the sample, add 20μL ChIP™ protein A+G magnetic beads, and incubate at 4 ℃ for 2 hours.
d) Precipitate magnetic beads on the magnetic frame at 4 ℃, and discard the supernatant after clarification for 1-2min.
e) Preheat the EB buffer at 65 ℃ in advance. Clean the magnetic beads with low salt and high salt buffer successively. Before cleaning, put all buffers on ice. During cleaning, wash three times with low salt and one time with high salt, and incubate at 4 ℃ for 5min.
f) Add 250μL preheated EB buffer. After upside down, incubate at 65 ℃ for 15min, and reverse every 5min (or vortex every 5min).
g) Place it on the magnetic frame for 1 min and collect the supernatant into a new EP tube.
h) Repeat steps f and g.

- **Reverse cross-linking and DNA recovery**

a) Thaw the nuclei sample.
b) 20μL 5M sodium chloride and 4μL Protease K was added into the experimental

sample and nuclei sample respectively.

c) The mixture was incubated at 65 °C for 6 hours or overnight.
d) 2μL RNase A was added into the experimental sample and nuclei sample respectively. Incubate at room temperature for one hour.
e) DNA Purification Kit is used to recover DNA.
